# Distribution of acetylcholinesterase (*Ace-1*^*R*^) target-site G119S mutation and resistance to carbamates and organophosphates in *Anopheles gambiae* sensu lato populations from Cameroon

**DOI:** 10.1186/s13071-022-05174-1

**Published:** 2022-02-14

**Authors:** Achille Jerome Binyang, Emmanuel Elanga-Ndille, Billy Tene-Fossog, Cyrille Ndo, Lynda Nouage, Tatiane Assatse, Yvan Fotso-Toguem, Raymond Tabue, Francis Zeukeng, Daniel Nguete Nguiffo, Josiane Etang, Flobert Njiokou, Charles S. Wondji

**Affiliations:** 1 Department of Medical Entomology, Centre for Research in Infectious Diseases (CRID), P.O. BOX 13591, Yaoundé, Cameroon; 2 Department of Animal Biology and Physiology, Faculty of Science, University of Yaoundé 1, P.O. Box 812, Yaoundé, Cameroon; 3 Vector Borne Diseases Laboratory of the Biology and Applied Ecology Research Unit (VBID-URBEA), Department of Animal Biology, Faculty of Science of the University of Dschang, Dschang, Cameroon; 4 Department of Parasitology and Microbiology, Centre for Research in Infectious Diseases (CRID), P.O. BOX 13591, Yaoundé, Cameroon; 5 Department of Biological Science, Faculty of Medicine and Pharmaceutical Sciences, University of Douala, P.O. Box 24157, Douala, Cameroon; 6 Ministry of Public Health, National Malaria Control Programme, P.O. Box 14386, Yaoundé, Cameroon; 7 National Reference Unit for Vector Control, The Biotechnology Centre, University of Yaoundé I, P.O. Box, 3851-Messa, Yaoundé, Cameroon; 8 Organisation de Coordination Pour La Lutte Contre Les Endémies en Afrique Centrale, BP 288, Yaoundé, Cameroun; 9 Department of Vector Biology, Liverpool School of Tropical Medicine, Pembroke Place, Liverpool, L3 5QA UK

**Keywords:** *Ace-1* G119S mutation, Insecticide resistance, *An. gambiae* s.l., Cameroon

## Abstract

**Background:**

Cameroon is considering the implementation of indoor residual spraying (IRS) as a complementary measure to control malaria in the context of high pyrethroid resistance in major malaria vectors. Non-pyrethroid insecticide classes such as organophosphates and carbamates may be utilized in IRS due to widespread pyrethroid resistance. However, the success of this strategy depends on good knowledge of the resistance status of malaria vectors to carbamates and organophosphates. Here, we assessed the susceptibility profile of *Anopheles gambiae* sensu lato with respect to carbamates and organophosphate and the distribution of the molecular mechanism underlying resistance to these insecticides.

**Methods:**

*Anopheles gambiae* s.l. mosquitoes were collected from nine settings across the country and bio-assayed with bendiocarb, propoxur and pirimiphos-methyl. The *Ace-1* target-site G119S mutation was genotyped using a TaqMan assay. To investigate the polymorphism in the *Ace-1* gene, a region of 924 base pairs in a sequence of the gene was amplified from both live and dead females of *An. gambiae* exposed to bendiocarb.

**Results:**

Pirimiphos-methyl induced full mortality in *An. gambiae* s.l. from all study sites, whereas for carbamates, resistance was observed in four localities, with the lowest mortality rate recorded in Mangoum (17.78 ± 5.02% for bendiocarb and 18.61 ± 3.86% for propoxur) in the southern part of Cameroon. *Anopheles coluzzii* was found to be the predominant species in the northern tropical part of the country where it is sympatric with *Anopheles arabiensis*. In the localities situated in southern equatorial regions, this species was predominant in urban settings, while *An. gambiae* was the most abundant species in rural areas. The G119S *Ace-1* target-site mutation was detected only in *An. gambiae* and only in the sites located in southern Cameroon. Phylogenetic analyses showed a clustering according to the phenotype.

**Conclusion:**

The occurrence of the *Ace-1* target-site substitution G119S in *An. gambiae* s.l. populations highlights the challenge associated with the impending deployment of IRS in Cameroon using carbamates or organophosphates. It is therefore important to think about a resistance management plan including the use of other insecticide classes such as neonicotinoids or pyrrole to guarantee the implementation of IRS in Cameroon.

**Graphical Abstract:**

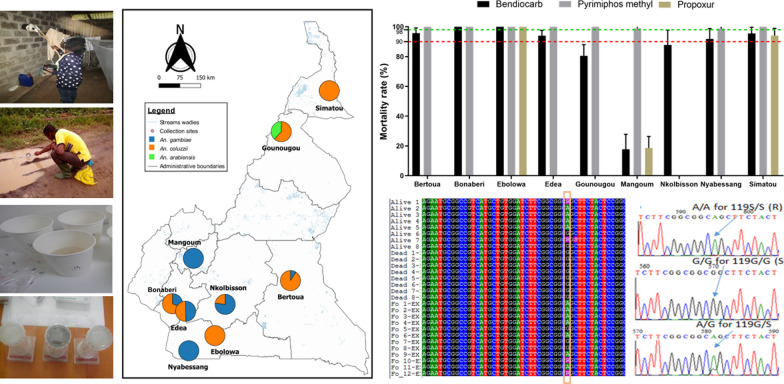

**Supplementary Information:**

The online version contains supplementary material available at 10.1186/s13071-022-05174-1.

## Background

Since the beginning of the 2000s, malaria control in Africa has been marked by intensive use of insecticide-based vector control strategies such as long-lasting insecticidal nets (LLINs) and indoor residual spraying (IRS) [1]. The rapid scaling up of these strategies has been so far the largest contributor to the decline recorded in malaria morbidity and mortality during the past two decades [1]. The World Health Organization (WHO) African Region in particular has achieved impressive reductions in its annual malaria mortality, from 840,000 deaths in 2000 to 602,000 in 2020 [2]. However, despite this significant progress, malaria remains a major public health concern in sub-Saharan Africa, with 10 countries listed among the 11 that account for approximately 70% of the world’s malaria burden [3].

Cameroon is among the 10 African countries most affected by malaria [3]. This disease remains the leading cause of morbidity and mortality in health facilities in the country. In 2018, malaria was responsible for 25.8% of health facility consultations and 14.3% of deaths [3, 4]. In the same year, hospital morbidity due to malaria was 31.5% among children under five and 22.3% among pregnant women [4]. Based on the severity of the problem, the Cameroonian government has made the fight against malaria a national priority highlighted in its national strategic planning documents, in particular the Health Sector Strategy 2016–2027 [5].

Over the past decade, the country has conducted three nationwide free LLIN distribution campaigns, notably in 2011, 2015 and 2019, resulting in 69% of the population possessing a LLIN and 73.4% of households with at least one LLIN for two persons [6]. Unfortunately, the exclusive use of pyrethroids for bed net impregnation has led to the development and rapid spread of resistance to this insecticide class in major malaria vectors across the country [7–11]. Pyrethroid resistance in malaria vectors has been reported to be one major factor that could compromise efforts put in place to reduce the malaria burden in sub-Saharan African countries [12]. To address this situation and to achieve its goal of reducing malaria-related morbidity and mortality by 60% from 2015 levels by 2023 [5], the Cameroonian government has introduced in its strategic plan against malaria, additional vector control interventions using insecticides of a different mode of action in combination with LLINs [5], as recommended by WHO [13]. For this purpose, with support from the President’s Malaria Initiative (PMI), the National Malaria Control Programme (NMCP) is currently preparing for the deployment of IRS in some districts of the country shortly [14]. Thus, many insecticides belonging to carbamates (bendiocarb), organophosphates (pirimiphos-methyl), neonicotinoids (clothianidin) and pyrrole (chlorfenapyr) classes are currently being tested to generate evidence that would guide the choice of suitable insecticide classes for IRS implementation in Cameroon.

The scaling up of carbamate- and organophosphate-based IRS using bendiocarb and pirimiphos-methyl in sub-Saharan Africa has been successful in several epidemiological settings and contributed to reducing malaria transmission rates during the last decade [15, 16]. However, as observed with pyrethroids, the use of these molecules in IRS also led to the development of insecticide resistance in major African malaria vectors [17–20].

In Cameroon, carbamates and organophosphates have never been used in public health, and very little information on the susceptibility profile of malaria vectors across the country is available. However, since 2011, limited data from a few localities indicates a reduction in mortality rates in *Anopheles gambiae* sensu lato mosquito populations after exposure to carbamates [21–23]. Moreover, we recently demonstrated for the first time that resistance to carbamate is associated with the acetylcholinesterase (*Ace-1*^*R*^) target-site mutation G119S in an *An. gambiae* population from one locality in a forested area in central Cameroon [23]. If this *Ace-1*^*R*^ mutation and other mechanisms such as overexpression of some detoxification enzymes spread across the country, it could compromise the effectiveness of the IRS strategy using these two insecticide classes. Therefore, to facilitate the rational deployment of such a strategy, information on the resistance to carbamates and organophosphates as well as the distribution of the *Ace-1*^*R*^ G119S mutation in malaria vectors across the country is vital. For this purpose, the present study aimed to characterize patterns of susceptibility to carbamate and organophosphate in natural populations of *An. gambiae* s.l. across Cameroon and to investigate the distribution of the G119S mutation nationwide.

## Methods

### Study sites and mosquito sampling

**Fig. 1:**
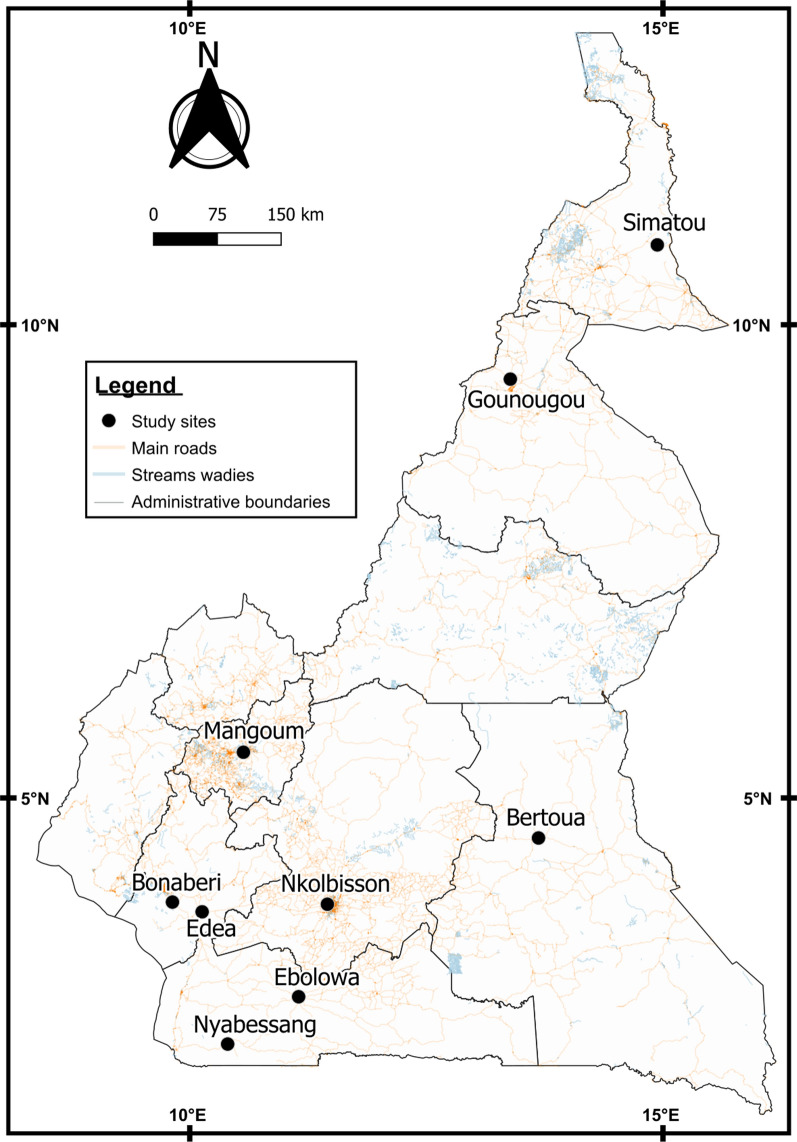
Map of Cameroon showing settings where wild *An. gambiae* s.l. mosquitoes were collected. The study sites where the samples were collected are represented by points. The map was constructed for this publication in QGIS 3.16 (https://www.qgis.org/fr/site/index.html) using country and region boundaries from GADM (https://gadm.org/download_country_v3.html)

*Anopheles gambiae* s.l. mosquitoes were collected between September and November 2019 in nine localities across the country (Fig. 1). The sites where mosquitoes were sampled in this study are all NMCP sentinel sites where entomological surveillance activities are currently implemented. These localities include the following:

- Seven localities from the southern equatorial forest zone in the south: Nkolbisson (3°87′N; 11°45′E), Bertoua (4°57′N; 13°68′E), Edéa (3°47′N; 10°07′E), Bonaberi (4°04’N; 9°39’E), Ebolowa (2°54' N, 11°9' E), Nyabessang (2°80'N, 10°25'E) and Mangoum (05°28’N, 10°35’E). These localities are characterized by a hot and humid climate with abundant rainfall and perennial malaria transmission for 7–12 months each year. The entomological inoculation rate in these areas is up to 100 infective bites/person/month [4].

- Gounougou (9°03′N, 13°43′E) in the tropical/Sudanian zone in the North region, characterized by seasonal malaria transmission, 4–6 months, with entomologic inoculation rate of 10 infective bites/person/month [4].

- Simatou (10°34′N, 14°30′E) in the Sahelian zone in the Far North region, characterized by a hot and dry tropical climate; malaria transmission is also seasonal, 1–3 months, with entomologic inoculation rate of 10 infective bites/person/month [4].

Mosquitoes were collected using two sampling methods:

Larval and pupae collection: This method was used in the localities of Nyabessang, Nkolbisson, Bertoua, Edéa, Bonaberi and Ebolowa, where densities of immature stages of *An. gambiae* s.l. were high. In each locality, larvae (L1 to L4) and pupae were collected in different types of breeding habitats including temporary or permanent puddles, brick pits, ponds and animal footprints using the “dipping” technique [24]. Larvae were transferred in well-labelled containers and then transported to the insectary at the Centre for Research in Infectious Diseases (CRID) in Yaoundé, where they were allowed to emerge into adults under standard insectary conditions (25 ± 2 °C temperature, 70–80% relative humidity, 12:12-h light/dark cycle). Emerged adults were maintained on 10% sucrose in cages.

Adult collection: This method was applied in the localities of Gounougou, Mangoum and Simatou, where *An. gambiae* s.l. productive breeding sites were found in low density. In each locality, after obtaining verbal consent from the household owners, indoor resting blood-fed or gravid adult female *Anopheles* mosquitoes were collected from the walls and roofs of different houses across the community between 6:00 AM and 10:00 AM using Prokopack electrical aspirators (John W. Hock, Gainesville, FL, USA). Captured mosquitoes were kept in paper cups in a cool place before transporting them to the insectary at CRID. Captured blood-fed female mosquitoes were kept until fully gravid and induced to lay eggs in individual 1.5-ml microcentrifuge tubes as described previously [25]. Eggs were stored at room temperature for up to 2 days and then transferred into small paper cups containing water to hatch. Larvae were transferred into bowls containing water and reared to F1 adults. Emerged adult mosquitoes were randomly mixed in cages and maintained on 10% sucrose under standard insectary conditions (temperature of 25 °C, relative humidity of 70–80% and 12:12-h light/dark cycle).

All adult mosquitoes obtained were morphologically identified and sorted by species according to the morphological identification keys of Gillies and De Meillon [26] and Gillies and Coetzee [27].

### Insecticide susceptibility bioassays

Insecticide susceptibility bioassays were performed using WHO insecticide-impregnated filter papers purchased from the Vector Control Research Unit (VCRU), University Sains Malaysia (http:/www. inreskit.usm.my). Non-blood-fed female *An. gambiae* s.l. of two to five days old were exposed to bendiocarb (0.1%), propoxur (1%) and pirimiphos-methyl (1%) using the WHO standard procedure for adult mosquitoes [28]. For each insecticide, four replicates of 20–25 mosquitoes per tube with four replicates were exposed to insecticide-impregnated papers for 1 h. Meanwhile, two batches of 20–25 mosquitoes exposed to untreated paper were used as a control. After the exposure period, mosquitoes were transferred to a clean holding tube, maintained at 28 ± 1 °C and 80 ± 10% relative humidity, and fed 10% sugar solution. The mortality was recorded 24 h post-exposure to insecticide-impregnated papers, and resistance status was evaluated according to the WHO criteria [28]. At the end of the test, live and dead mosquitoes were stored separately in silica gel and kept at −20 °C for molecular identification and resistance gene screening.

### Molecular identification of *An. gambiae* s.l.

For each insecticide, the genomic DNA of both live and dead mosquitoes after bioassays was extracted using the DNA extraction protocol described by Livak [29]. This DNA was used to identify sibling species of the *Anopheles gambiae* complex following the SINE PCR as previously described by Santolamazza et al. [30]. For each locality, mosquitoes were identified and separately sorted to *An. gambiae*, *An. coluzzii* and *An. arabiensis*.

### *Ace-1* G119S mutation genotyping

The presence of the G119S mutation in each member of the *An. gambiae* complex was screened using the TaqMan real-time PCR assay using Agilent Mx3005 Real-Time PCR thermocycler (Santa Clara, CA, USA) following the protocol described by Bass and colleagues [31]. For each sample, the reaction was conducted in a total volume of 10 µl comprising 5 µl SensiMix (Bioline, London, UK), 0.25 µl of 40× Probe Mix coupled to allelic-specific primers [*Ace-1* forward (5′-GGC CGT CAT GCT GTG GAT-3′); *Ace-1* reverse (5′-GCG GTG CCG GAG TAG A-3′); ACE1-VIC (5′-TTC GGC GGC GGC T-3′); ACE1-6-FAM (5′-TTC GGC GGC AGCT-3′)], 4.25 µl of dH20 and 1 µl of genomic DNA. Thermal cycling conditions were an initial 10 min at 95 °C, followed by 40 cycles each of 92 °C for 15 s and 60 °C for 1 min. Probes were labelled with two different fluorescent dyes FAM™ and HEX™ and were used to detect the resistance mutant (RR) and the wild-type susceptibility (SS) alleles, respectively. After amplification, genotypes were scored from bidirectional scatter plots of results produced by Agilent Mx3005 v4.10 software.

### *Ace-1* sequencing

To investigate the polymorphism in the *Ace-1* gene, a region of 924 base pairs (bp) in a sequence of the gene, encompassing exons 4–6 (VectorBase AgamP3 annotation, AGAP001356; G119S position in exon 5 corresponding to the third coding exon) was amplified from both live and dead females of *An. gambiae* exposed to bendiocarb. As previously described by Elanga-Ndille et al. [23], each PCR was conducted on a total volume of 50 μl containing 10 pmol of each primer Ex2Agdir1 (5′ AGG TCA CGG TGA GTC CGTACG A 3′) and Ex4Agrev2 (5′ AGG GCG GAC AGC AGA TGC AGC GA 3′), 10 mM dNTPs, ddH2O, 5× Phusion High-Fidelity buffer and 1 U of Phusion Taq polymerase (Fermentas, Burlington, ON, Canada). The PCR conditions were an initial cycle at 98 °C for 4 min, followed by 35 cycles of 98 °C for 30 s, 64 °C for 15 s and 72 °C for 30 s, with a final extension at 72 °C for 5 min. After purification using the QIAquick purification kit (Qiagen, Hilden, Germany), the PCR products were sequenced directly using the primers Ex2Agdir1 and Ex4Agrev2 to confirm the presence of the G119S mutation and assess the signature of selection at this *Ace-1* in each locality. The ClustalW program [32] as implemented in BioEdit software [33] was used to align the DNA sequence with a consensus sequence from the Kisumu strain (*An. gambiae* strain susceptible to all insecticides) exported from VectorBase (gene ID: AGAP001356) and used as reference. The polymorphism analysis was performed using DnaSP v5.10 [34], while MEGA 10.1.0 [35] was used to build a maximum-likelihood tree from the aligned sequences after length equalization using the Tamura 3-parameter model selected after performing the model test. A haplotype network was also constructed using the TCS program [36] and tcsBU [37].

### Statistical analysis

Following exposure of mosquito populations to insecticides, populations with mortality rates less than 90% were considered to be indicative of resistance and those with mortality rates greater than 98% were indicative of susceptibility (Additional file 1). Mortality rates of 90–98% suggested probable resistance that needed to be confirmed [28]. After PCR analysis, *Ace-1* genotype distributions were recorded in an Excel datasheet (Microsoft Office 2016; Microsoft Corporation, Redmond, WA, USA), and Fisher’s exact test was performed using GraphPad Prism version 7.00 software (GraphPad Software Inc., La Jolla, CA, USA). Allelic frequencies were calculated using the formula ƒ(R) = (2*n*.RR + *n*.RS)/2*N*, where *n* is the number of mosquitoes of a given genotype, and *N* is the total number of mosquitoes analysed.

## Results

### Insecticide resistance profile of *An. gambiae* s.l. populations from study sites

**Fig. 2:**
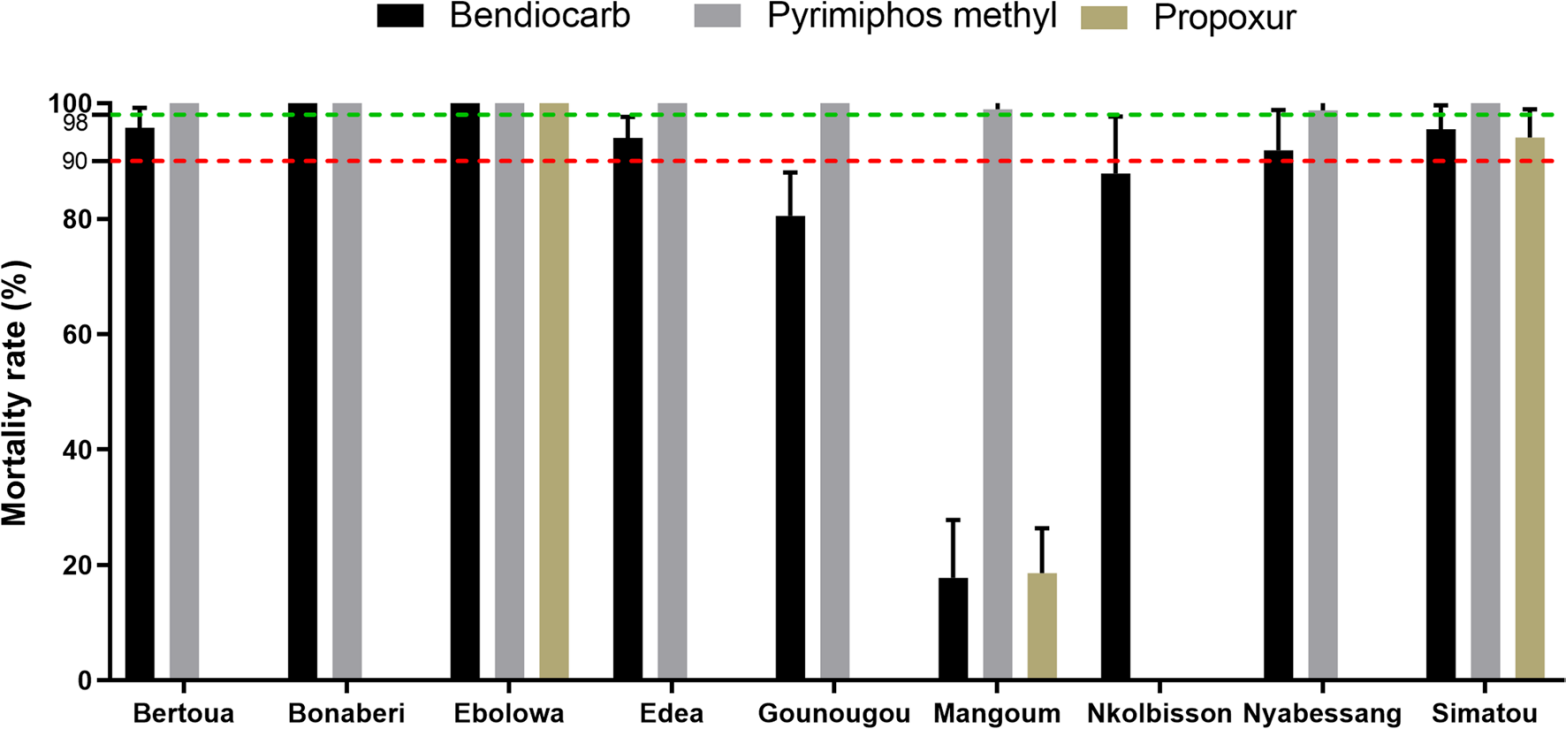
Susceptibility profile of *An. gambiae* s.l. populations from Cameroon with respect to bendiocarb, propoxur and pirimiphos-methyl. Mortality rates were recorded 24 h post-exposure to insecticides. Data are shown as mean ± standard error of the mean (SEM). The red dotted line represents threshold mortality for WHO resistant population, whereas the green dotted line represents threshold mortality for the WHO susceptible population

A total of 2188 adult female mosquitoes from the nine study sites were exposed to both carbamates (bendiocarb and propoxur) and organophosphates (pirimiphos-methyl). The profile revealed that all nine populations of *An. gambiae* s.l. were susceptible to pirimiphos-methyl, as they exhibited 100% mortality 24 h post-exposure. Concerning bendiocarb, full susceptibility (100% mortality rate) was observed in *An. gambiae* s.l. populations from Bonaberi and Ebolowa, while probable resistance was observed in Bertoua (98.97 ± 2.21%), Edéa (94 ± 3.02%), Nyabessang (91.80 ± 3.52%) and Simatou (95.55 ± 3.22%) populations. However, resistance to bendiocarb was observed in Gounougou (80.45 ± 4.97%), Mangoum (17.78 ± 5.02%) and Nkolbisson (87.80 ± 4.97%) *An. gambiae* s.l. populations. Propoxur bioassays were carried out only on *An. gambiae* s.l. populations from Ebolowa, Simatou and Mangoum. Full susceptibility to this insecticide was observed for the mosquito population from Ebolowa, and probable resistance (94.05 ± 5.95%) and resistance were observed in Simatou and Mangoum (18.61 ± 3.86%), respectively (Fig. 2).

### Distribution of mosquito species

**Fig. 3:**
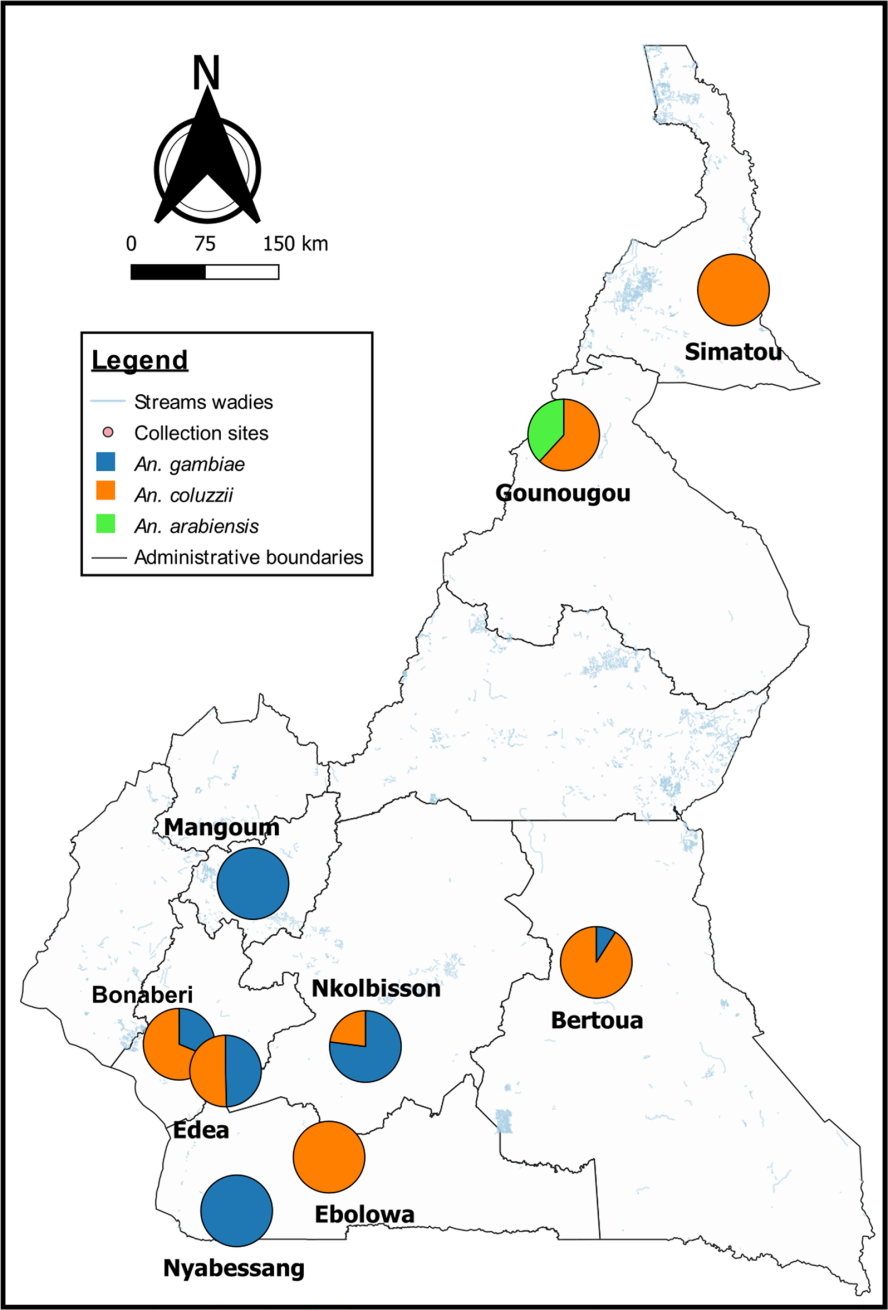
Map of Cameroon showing the relative frequency and the distribution of mosquito species within *An. gambiae* complex across the country

For each locality, live and dead mosquitoes after bioassays were used for molecular species identification within the *An. gambiae* complex. Results of the analyses showed that *An. coluzzii*, *An. gambiae* and *An. arabiensis* were the three mosquito species identified among the *An. gambiae* complex in the nine surveyed localities, with a variable distribution (Fig. 3, Additional file 2). Data showed that *An. coluzzii* was found in all the study sites except in Mangoum and Nyabessang. This species was found to be predominant in Simatou (98%), Gounougou (62%), Bonaberi (65%), Bertoua (81%) and Ebolowa (100%). *Anopheles gambiae* species, on its side, was identified only in the sites located in the southern forest part of the country, except in Ebolowa. This species was predominant in Mangoum (100%), Nyabessang (100%) and Nkolbisson (77%). In Edéa, *An. gambiae* was found in the same proportion (50%) as *An. coluzzii*, whereas in Bonaberi it was less abundant (35%). Concerning *An. arabiensis*, it was found only in Gounougou in the northern part of the country. In this locality, this species was found sharing its breeding sites with *An. coluzzii* (Fig. 3).

### Distribution of *Ace-1* G119S mutation in *An. gambiae* complex in study sites

*Ace-1* G119S genotypes were characterized in a total of 1273 females of *An. gambiae* s.l. mosquitoes from all study sites including those both dead and alive after exposure to bendiocarb, propoxur and pirimiphos-methyl. The *Ace-1* G119S mutation allele was detected only in *An. gambiae* from Bertoua, Edéa, Nyabessang, Nkolbisson and Mangoum (Table 1). The frequency of the *Ace-1*^*R*^ allele varied from 3.00% to 41.18% in Edéa and Bertoua, respectively (Table 1). Mosquitoes carrying the resistance allele were significantly more likely to survive after exposure to bendiocarb than those with the susceptibility allele (*P* < 0.0001, OR = 155; 95% CI = 35.5–677.08). Homozygote-resistant mosquitoes displayed a higher ability to survive than homozygote-susceptible mosquitoes (*P* < 0.0001, OR = 148, 95% CI = 41.77–524.94). Also, heterozygote-resistant mosquitoes were significantly more likely to survive than homozygote-susceptible mosquitoes (*P* < 0.000.1, OR = 45; 95% CI = 5.12–398;). No difference was observed between homozygote-resistant and heterozygote-resistant mosquitoes (*P* = 0.3, OR = 3.27; 95% CI = 0.29–36.64).

**Table 1:** Frequencies of the *Ace-1*^*R*^ allele and genotypes in the *An. gambiae* s.l. samples collected in 2019 from nine localities in Cameroon

Locality	Number analysed	Species (number)	*Ace-1* mutation genotypes			Allelic frequency	
			RR	RS	SS	R	S
Bertoua	188	*An. gambiae* (17)	7/17 (41.18%)	0	10/17 (58.82%)	0.41	0.59
		*An. coluzzii* (171)	0	0	171/171 (100%)	0	1
Ebolowa	150	*An. coluzzii* (150)	0	0	150/150 (100%)	0	1
Edéa	155	*An. gambiae* (67)	2/67 (3%)	0	65/67 (97%)	0.03	0.97
		*An. coluzzii* (88)	0	0	88 (100%)	0	1
Bonaberi	63	*An. gambiae* (22)	0	0	22 (100%)	0	1
		*An. coluzzii* (41)	0	0	41 (100%)	0	1
Gounougou	178	*An. arabiensis* (68)	0	0	68 (100%)	0	1
		*An. coluzzii* (110)	0		110 (100%)	0	1
Mangoum	252	*An. gambiae* (252)	88/252 (35%)	6/252 (2.3%)	158/252 (62.7%)	0.36	0.64
Nyabessang	145	*An. gambiae* (145)	7/145 (4.8%)	0	138 (95.2%)	0.05	0.95
Nkolbisson	61	*An. gambiae* (47)	9/47 (19.2%)	0	38/47 (80.8%)	0.15	0.85
		*An. coluzzii* (14)	0	0	14 (100%)	0	1
Simatou	81	*An. coluzzii* (81)	0	0	81(100%)	0	1

*RR* homozygote-resistant, *RS* heterozygote-resistant, *SS* homozygote-susceptible

### Genetic diversity and phylogenetic analysis of *Ace-1* in *An. gambiae* from Cameroon

To confirm the presence of the 119S allele and assess the genetic diversity of the *Ace-1* gene, a region of 924 bp of this gene was sequenced from 71 *An. gambiae* mosquitoes (44 dead and 27 alive after exposure to bendiocarb) (Table 2). After sequence alignment, a region of 586 bp was commonly aligned, and a G-to-A substitution at position 394, corresponding to the 119th codon, was observed in seven sequences (one from Bertoua, four from Mangoum and two from Edéa) in comparison with the reference sequence from the susceptible Kisumu strain. Also, 20 heterozygote mosquitoes (two from Bertoua, seven from Mangoum, six from Nkolbisson and five from Nyabessang) were detected (Additional file 1). Furthermore, no substitution was detected in any of the sequences from dead mosquitoes across all study sites.

**Table 2:** Summary statistics for polymorphism in *Ace-1* gene including the G119S mutation in dead and live *An. gambiae* mosquito populations from Cameroon after exposure to carbamates

	2n	S	NSyn	Syn	h	hd	π	D	D*
Alive	54	12	1	8	23	0.888	0.004	−0.290 ns	−0.569 ns
Dead	92	31	5	20	38	0.927	0.005	−1.519 ns	−1.436 ns
Total	146	32	5	21	55	0.950	0.006	−1.210 ns	−1.825 ns

*2n* number of sequences, *S* number of polymorphic sites, *Syn* synonymous substitution, *NSyn* non-synonymous substitution, *h* number of haplotypes, *hd* haplotype diversity, *π* nucleotide diversity, *D* Tajima’s statistics, D* Fu and Li’s statistics (the asterisk indicates without an outgroup), *ns* not significant

**Fig. 4:**
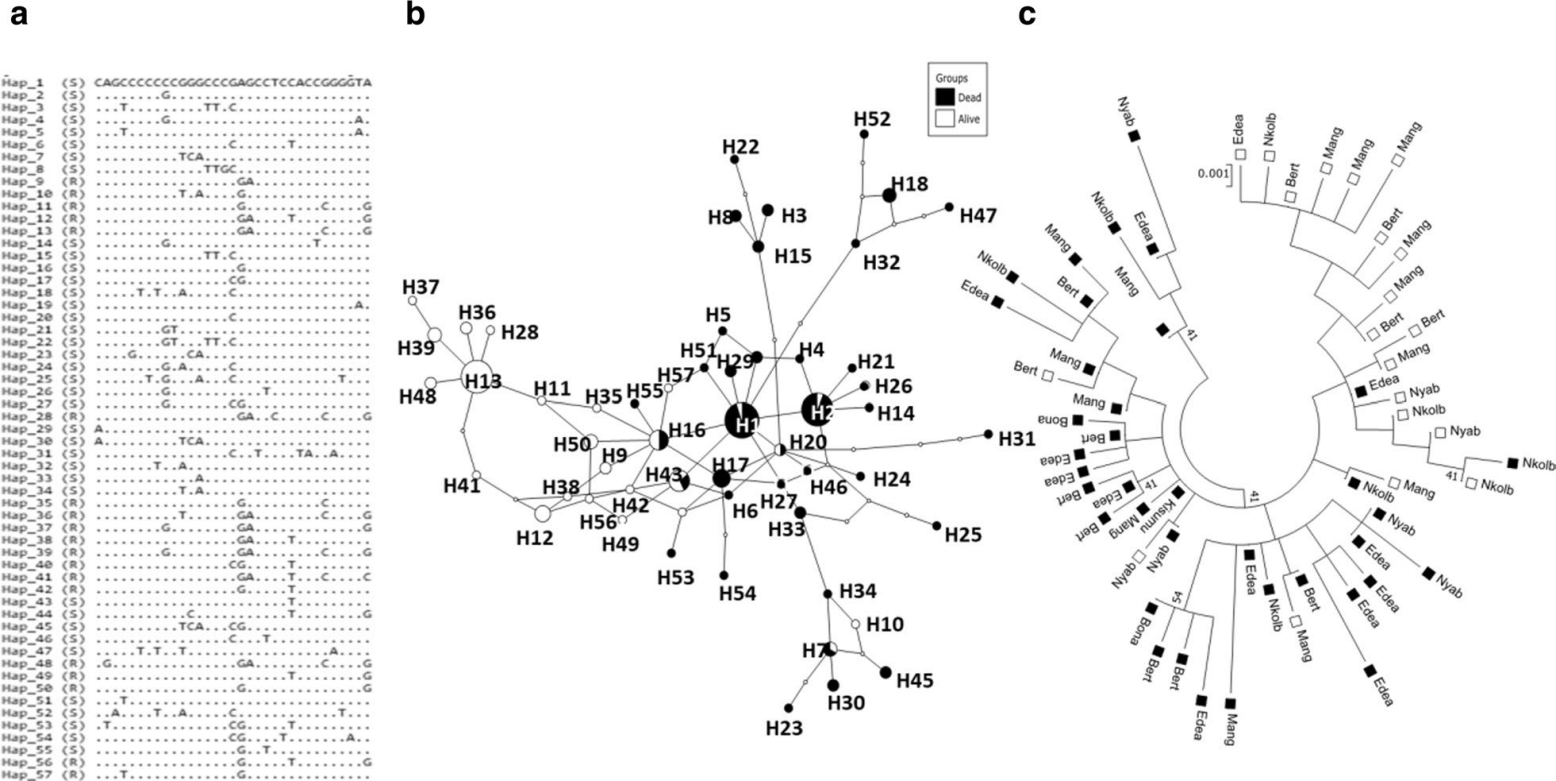
Polymorphism patterns of *Ace-1* gene from direct sequencing. **a** Polymorphic sites and haplotypes detected. Haplotypes are labelled with S (susceptible) or R (resistant). **b** The Templeton, Crandall and Sing (TCS) haplotype network shows the resistance and susceptibility haplotype clusters. Lines connecting haplotypes and each node represent a single mutation event. **c** Maximum-likelihood phylogenetic tree of *Ace-1* gene supporting the clustering of haplotypes according to the mosquito resistance status

Analysis of the polymorphism pattern of the *Ace-1* gene showed 32 polymorphic sites including 12 and 31 for live and dead individuals, respectively (Table 2). The number of haplotypes (38 vs 23) and haplotype diversity (0.927 vs 0.888) were higher for dead than live mosquitoes. Among the 55 different haplotypes recorded, only 27 had the 119S allele (Fig. 4a). These haplotypes showed a slight trend to clustering in the haplotype network (Fig. 4b), as well as on a maximum likelihood phylogenetic tree (Fig. 4c) according to their genotype, with those containing the 119S allele separate from those harbouring the susceptible ones. The genetic diversity was very low in mosquito populations from each locality as well as in the overall population (Table 3). Four non-synonymous (one in live and five dead mosquitoes) and 21 synonymous mutations were observed in the total population. (Table 3). Overall, Tajima’s D and the Fu and Li index were negative but not statistically significant. The haplotype network and maximum likelihood phylogenetic tree generated show haplotype clustering associated with either dead or live mosquitoes.

**Table 3:** Summary statistics for polymorphism in the *Ace-1* gene including the G119S mutation in *An. gambiae* mosquito populations from six localities in Cameroon

	2n	S	NSyn	Syn	h	hd	π	D	D*
Bertoua	22	15	1	11	13	0.935	0.006	−0.233 ns	−1.170 ns
Bonaberi	12	5	1	4	4	0.697	0.002	−0.987 ns	−1.531 ns
Edéa	30	21	3	16	18	0.939	0.005	−1.268 ns	−0.993 ns
Mangoum	38	17	3	13	22	0.943	0.006	−0.477 ns	0.478 ns
Nkolbisson	20	15	1	12	13	0.905	0.006	−0.199 ns	−0.528 ns
Nyabessang	24	19	2	12	16	0.949	0.006	−1.265 ns	−1.686 ns
Total	146	32	5	21	55	0.950	0.006	−1.210 ns	−1.825 ns

*2n* number of sequences, *S* number of polymorphic sites, *Syn* synonymous substitution, *NSyn* non-synonymous substitution, *h* number of haplotypes, *hd* haplotype diversity, *π* nucleotide diversity, *D* Tajima’s statistics, D* Fu and Li’s statistics (the asterisk indicates without an outgroup), *ns* not significant

## Discussion

To manage the challenge from pyrethroid resistance and to reduce the burden of malaria, Cameroon aims to implement an IRS strategy in some districts across the country. For this purpose, the NMCP is currently testing non-pyrethroid insecticides including carbamates and organophosphates, both used in the last decade for IRS across the continent, contributing to reducing the malaria burden [38]. However, because a reduction in mortality rate after exposure to carbamate was reported in *Anopheles* populations from a few localities [21–23], and because the *Ace-1*^*R*^ G119S mutation that confers resistance to both carbamate and organophosphate was recently detected in the country [23], nationwide information on the susceptibility profile of *An. gambiae* s.l. populations as well as the distribution of *Ace-1* mutation was needed to guide the deployment of any carbamate- or organophosphate-based IRS intervention.

### Insecticide susceptibility

*Anopheles gambiae* s.l. populations from all nine localities studied were found fully susceptible to pirimiphos-methyl, belonging to the organophosphates insecticide class. Previously, several studies have recorded full susceptibility to organophosphates in *An. gambiae* s.l. populations from various other locations in Cameroon [9, 23, 39–42]. This result suggests that organophosphate compounds such as pirimiphos-methyl could be used for IRS in most of the locations in Cameroon where pyrethroid-resistant populations are found. Concerning carbamates, the susceptibility profile in *An. gambiae* s.l. mosquitoes was very heterogeneous across the country. The full susceptibility observed in Bonaberi and Ebolowa contrasts with the suspected resistance in the Edéa, Simatou, Nyabessang and Bertoua *An. gambiae* s.l. populations, on one hand, and with the confirmed resistance in the populations from Gounougou, Nkolbisson and Mangoum on other hand. Results of the present study confirm the increasing trends of carbamate resistance in *An. gambiae* s.l. populations from Cameroon as previously reported in other studies [8, 22, 23, 43]. The origin of the selection of this resistance remains unknown, although it can be hypothesized that it has resulted from agricultural use of carbamate-based pesticides, as no public health control strategy has been implemented in Cameroon with this class of insecticides [5, 14]. This hypothesis is supported by the previous study of Antonio-Nkondjio et al. [43], who demonstrated that mosquitoes originating from cultivated sites were more resistant to bendiocarb than those collected elsewhere. Moreover, this hypothesis is reinforced by the higher level of resistance to carbamate observed in the locality of Mangoum, which among the nine study sites is known to experience the use of large amounts of pesticides for intensive vegetable cultivation. Furthermore, the variability observed in the susceptibility of *An. gambiae* s.l. to carbamates is likely due to geographical heterogeneity in selection pressure to which mosquitoes are exposed [43]. The occurrence of resistance to carbamates could have a detrimental effect on the efficacy of a bendiocarb and/or propoxur-based strategy used as an alternative for malaria vector control in Cameroon. Therefore, it is important to follow up on the evolution of observed carbamate resistance and to understand the factors driving the development of this resistance, its underlying mechanisms and its distribution across the country.

### Molecular identification of *An. gambiae* species across the nine surveyed localities

Molecular analyses showed that *An. arabiensis*, *An. gambiae* and *An. coluzzii* were the only members of the *An. gambiae* complex identified in the study sites. However, several disparities were observed in the distribution of these species across the country. Indeed, *An. arabiensis* was found only in the northern region and *An. gambiae* only in sites located in the southern region, while *An. coluzzii* was present in both the northern and southern parts of the country. This geographical distribution pattern indicates that both *An. arabiensis* and *An. gambiae* remain localized in their ecological niche, as described in previous studies [44, 45]. However, the high frequency of *An. coluzzii* compared to *An. arabiensis* in the northern part of the country is in line with previous studies which hypothesized that this species is progressively becoming the dominant vector species in Sudano-Sahelian savannah zones [41, 46]. Nevertheless, longitudinal studies are needed to further establish this trend. This is in contrast to the previous observations of *An. arabiensis* and *An. gambiae* (former S form) as the predominant species of the *An. gambiae* complex in the northern regions of Cameroon [44, 45]. This adaptation of *An. coluzzii* could have been favoured by anthropogenic activities, such as farming. Irrigated rice cultivation is copiously observed in northern Cameroon, and this activity creates semi-permanent and permanent water bodies known to be preferential breeding sites for *An. coluzzii* [47]. Species distribution in the southern region showed that *An. gambiae* and *An. coluzzii* are predominant in rural (Mangoum, Nyabessang, Nkolbisson) and urban (Ebolowa, Douala, Bertoua) areas, respectively. This is in line with findings supporting the high adaptation capacity of *An. coluzzii* in the urban environment, while *An. gambiae* prefers rural habitats [45, 47, 48].

### Distribution of *Ace-1*^*R*^ mutation in *An. gambiae* s.l. populations

Resistance to carbamates in *An. gambiae* s.l. mosquitoes in some localities in the southern region of Cameroon was recently reported to be significantly associated with the presence of *Ace-1* mutation (G119S) due to a substitution of glycine by the serine in codon 119 of the gene [23]. In the present study, analyses to investigate the presence of the *Ace-1* G119S mutation in *An. gambiae* s.l. mosquitoes collected across the country show that the resistance allele was detected only in localities situated in the southern regions of Cameroon and not in the northern regions. Furthermore, the high resistance to carbamate was associated with high frequencies of the 119S allele as observed in Mangoum and Bertoua. Our findings here confirm the implication of *Ace-1*^*R*^ mutation for the development of resistance to carbamates in *An. gambiae* s.l. from Cameroon as already recently reported [23]. The 119S mutant allele was detected only in *An. gambiae* species even in localities where this species was found in sympatry with *An. coluzzii*. This overlapping distribution between *An. gambiae* and the *Ace-1*^*R*^ mutation seems to justify the nationwide resistance profile observed in this study, with resistance detected mostly in locations where *An. gambiae* was present. The detection of the *Ace-1* G119S mutation only in *An. gambiae* is in contrast to a previous study of Djogbenou and colleagues in West Africa, which speculated that the *Ace-1* G119S mutation first occurred in *An. coluzzii* (former M form) and not in *An. gambiae* [49]. This mutation has not resulted in expected cross-resistance to organophosphates because G119S had a greater impact on carbamate than organophosphate resistance in *An.* *gambiae* as suggested by Djogbenou et al. [49]. This mutation occurs with higher frequency of homozygous resistance (R/R) than heterozygous resistance, suggesting a deviation from Hardy–Weinberg equilibrium, as already observed in a recent study in Cameroon [23] and others in West Africa. Furthermore, the low frequency of resistance observed in almost all study sites except in Mangoum suggests that this mutation recently occurred and is still spreading in *An. gambiae* s.l. populations across Cameroon. This signature of a recent expansion of the resistance allele in *An. gambiae* populations from Cameroon is confirmed by the negative values observed for Tajima’s test. The higher haplotype diversity in dead mosquitoes compared to live ones suggests a selective sweep acting on the *Ace-1* gene in carbamate-resistant mosquitoes. This is similar to signatures of selection observed for other resistance loci in *An. gambiae* for both target-site and metabolic resistance [50] as well as in *Anopheles funestus* for GST [51] and P450-based [52] metabolic resistance mechanisms.

## Conclusion

This study revealed that *An. gambiae* s.l. populations from Cameroon were fully susceptible to organophosphates but exhibited variable susceptibility to carbamates across the country. The level of carbamate resistance was associated with the presence and the frequency of the *Ace-1* G119S mutation, which was detected only in *An. gambiae* species and not in *An. coluzzii* or *An. arabiensis*. The distribution of this mutation overlapped with that of *An. gambiae* and was found only in the southern part of the country. Overall, the organophosphate insecticide class would be more suitable than carbamates for IRS in Cameroon, especially in the southern part of the country. However, because the potential spread of the *Ace-1* G119S mutation could compromise the efficacy of such a strategy, periodic updates of its distribution and data on resistance of *An. gambiae* s.l. mosquitoes should be performed to guide the implementation of IRS strategies in Cameroon.

## Supplementary Information

**Additional file 1**. Sequence alignment of the *Ace-1* gene at the G119S point mutation in live and dead *An. gambiae* mosquitoes 24 h after exposure to carbamate.

**Additional file 2**. Number of *An. gambiae* s.l. mosquitoes identified among those alive and dead after exposure to insecticides.

## Data Availability

Data are available from the corresponding author upon reasonable request.
