# Supplementary material for: Distribution of acetylcholinesterase (Ace-1R) target-site G119S mutation and resistance to carbamates and organophosphates in Anopheles gambiae sensu lato populations from Cameroon
Source: Parasit Vectors. 2022 Feb 14;15:53. doi: 10.1186/s13071-022-05174-1 (PMC8842952; doi:10.1186/s13071-022-05174-1)

## Slide 1
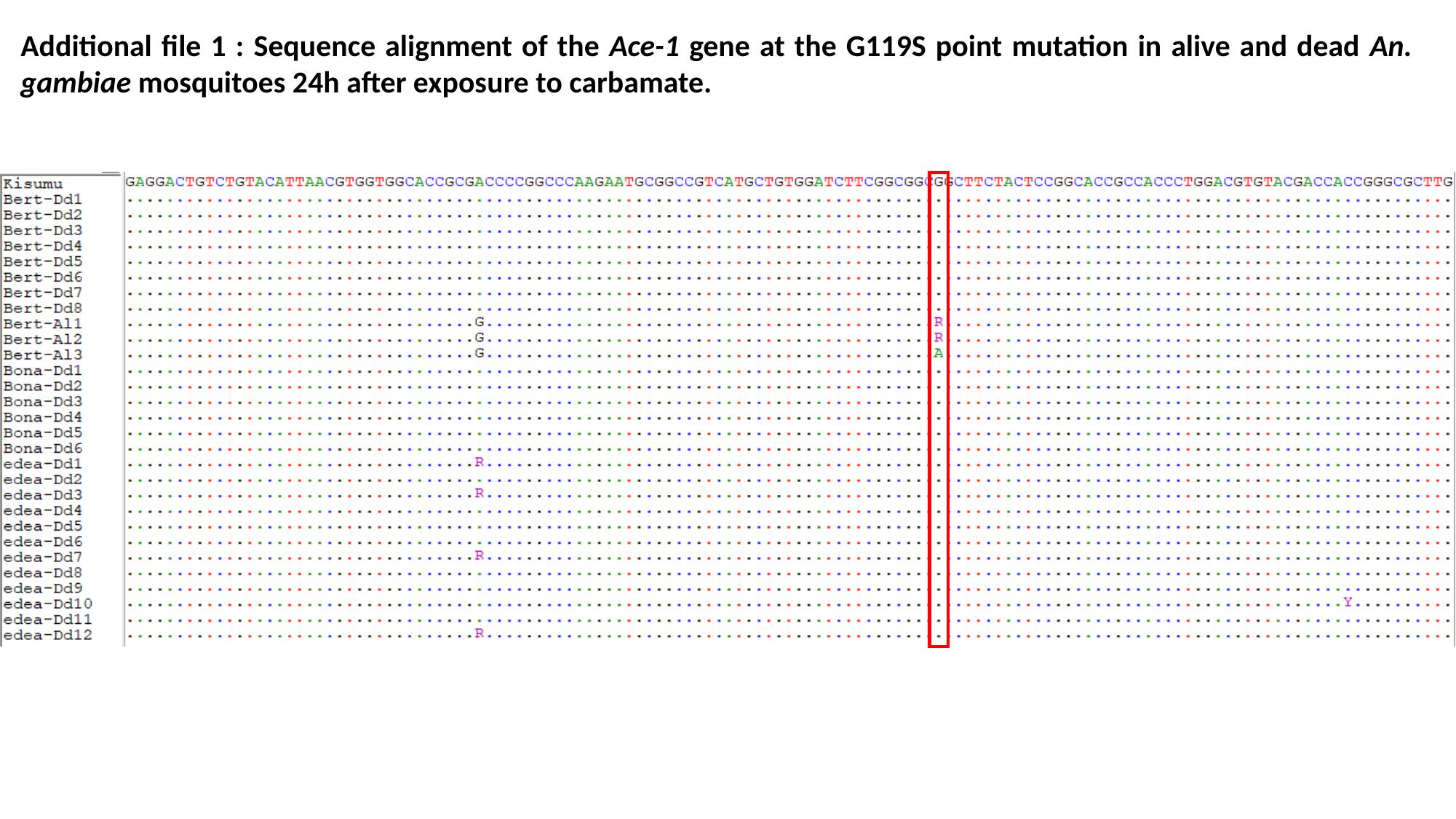

Additional file 1 : Sequence alignment of the Ace-1 gene at the G119S point mutation in alive and dead An. gambiae mosquitoes 24h after exposure to carbamate.

## Slide 2
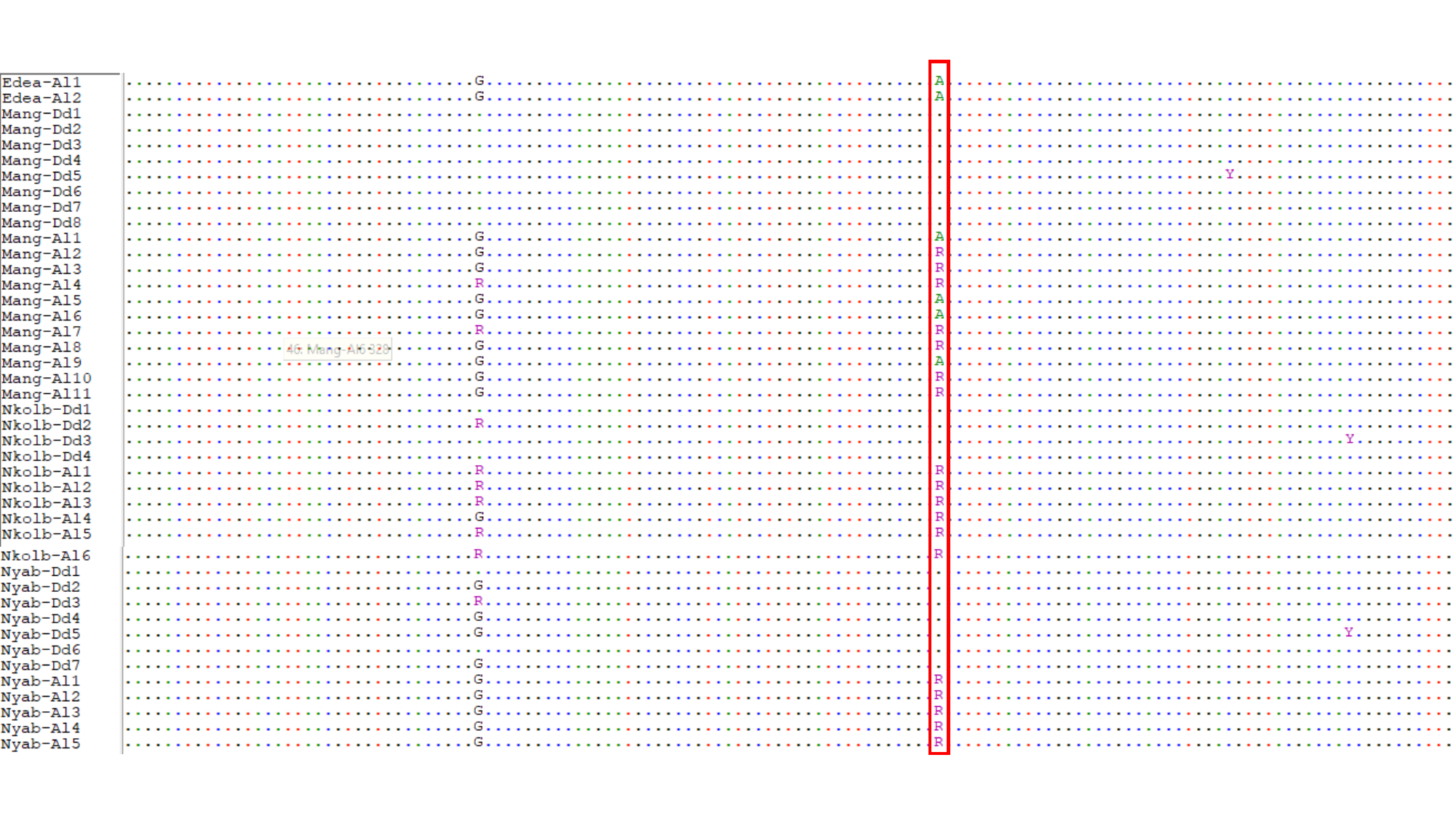

Supplement: Supplementary file 1 — Additional file 1. Sequence alignment of the Ace-1 gene at the G119S point mutation in live and dead An. gambiae mosquitoes 24 h after exposure to carbamate. [file 13071_2022_5174_MOESM1_ESM.pptx]
