# Supplementary material for: Distribution of acetylcholinesterase (Ace-1R) target-site G119S mutation and resistance to carbamates and organophosphates in Anopheles gambiae sensu lato populations from Cameroon
Source: Parasit Vectors. 2022 Feb 14;15:53. doi: 10.1186/s13071-022-05174-1 (PMC8842952; doi:10.1186/s13071-022-05174-1)
Supplement: Supplementary file 2 — Additional file 2. Number of An. gambiae s.l. mosquitoes identified among those alive and dead after exposure to insecticides. [file 13071_2022_5174_MOESM2_ESM.pptx]

## Slide 1
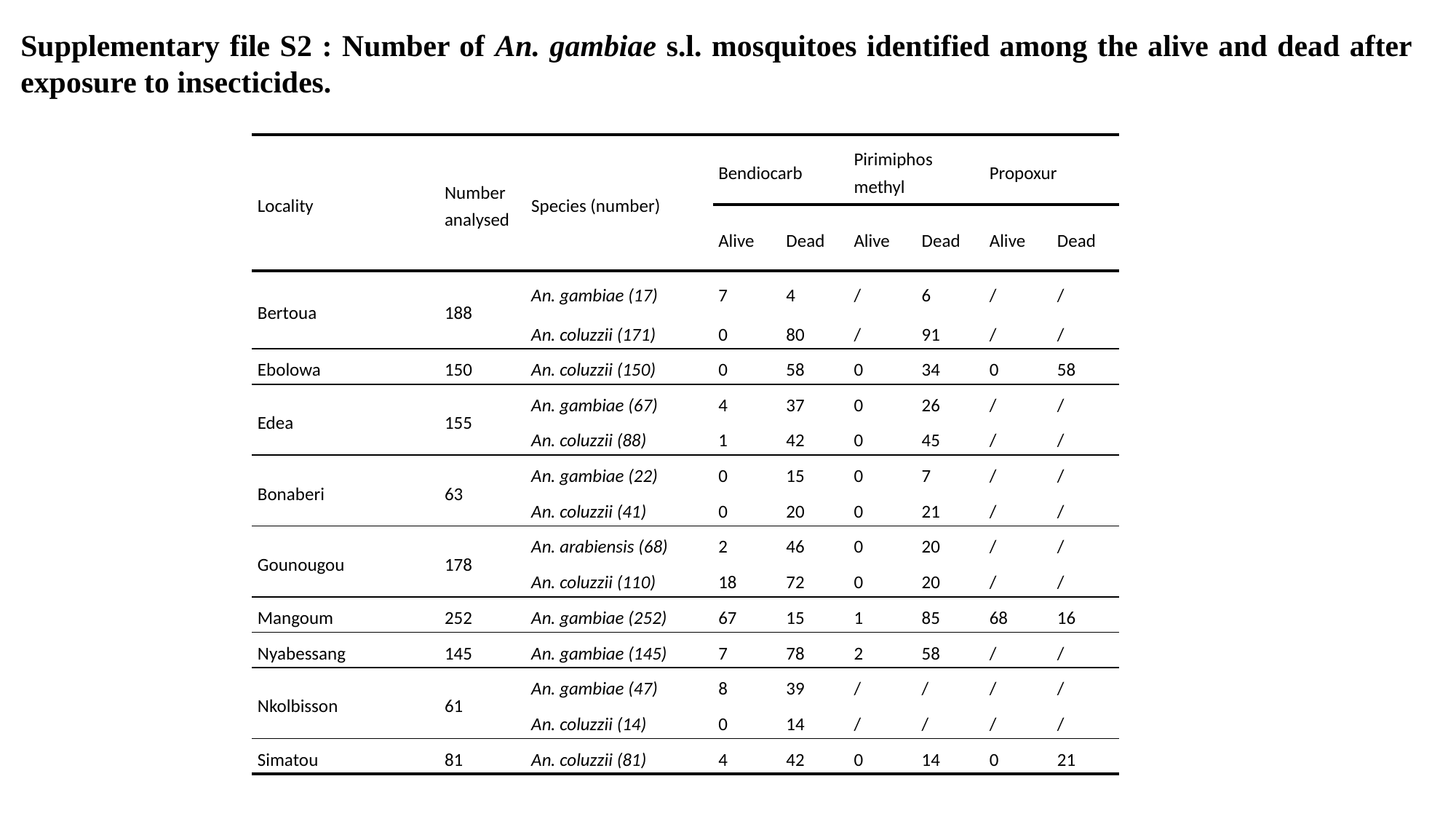

Supplementary file S2 : Number of An. gambiae s.l. mosquitoes identified among the alive and dead after exposure to insecticides.
| Locality | Number analysed | Species (number) | Bendiocarb | | Pirimiphos methyl | | Propoxur | |
| --- | --- | --- | --- | --- | --- | --- | --- | --- |
| | | | Alive | Dead | Alive | Dead | Alive | Dead |
| Bertoua | 188 | An. gambiae (17) | 7 | 4 | / | 6 | / | / |
| | | An. coluzzii (171) | 0 | 80 | / | 91 | / | / |
| Ebolowa | 150 | An. coluzzii (150) | 0 | 58 | 0 | 34 | 0 | 58 |
| Edea | 155 | An. gambiae (67) | 4 | 37 | 0 | 26 | / | / |
| | | An. coluzzii (88) | 1 | 42 | 0 | 45 | / | / |
| Bonaberi | 63 | An. gambiae (22) | 0 | 15 | 0 | 7 | / | / |
| | | An. coluzzii (41) | 0 | 20 | 0 | 21 | / | / |
| Gounougou | 178 | An. arabiensis (68) | 2 | 46 | 0 | 20 | / | / |
| | | An. coluzzii (110) | 18 | 72 | 0 | 20 | / | / |
| Mangoum | 252 | An. gambiae (252) | 67 | 15 | 1 | 85 | 68 | 16 |
| Nyabessang | 145 | An. gambiae (145) | 7 | 78 | 2 | 58 | / | / |
| Nkolbisson | 61 | An. gambiae (47) | 8 | 39 | / | / | / | / |
| | | An. coluzzii (14) | 0 | 14 | / | / | / | / |
| Simatou | 81 | An. coluzzii (81) | 4 | 42 | 0 | 14 | 0 | 21 |
